# Vascular stiffness and healthy arterial aging in older patients with optimal blood pressure

**DOI:** 10.1590/2175-8239-JBN-2022-0123en

**Published:** 2023-01-13

**Authors:** Alessandra Ferreira Mendes Jiticovski, Denis Fabiano Souza, Ercilhana Gonçalves Batista Freitas, Cléria Rodrigues Ferreira, Cristiane de Sousa Pereira, Romário Divino Vilarinho Galvão, Walkiria de Almeida Martins Santos, Erick P. de Oliveira, Sebastião Rodrigues Ferreira

**Affiliations:** 1 Universidade Federal de Uberlândia, Faculdade de Medicina, Uberlândia, MG, Brazil.

**Keywords:** Age, Vascular stiffnes, Heart Disease Risk Factor, Pulse Wave Analysis, Envelhecido, Rigidez Vascular, Fatores de Risco para Doença Cardíaca, Análise de Onda de Pulso

## Abstract

**Introduction:**

Pulse wave velocity is used to diagnose central arterial stiffness (CAS) and quantify healthy vascular aging (HVA).

**Objective:**

To evaluate the CAS and HVA in elderly patients with systemic blood pressure levels classified as optimal/normal.

**Methods:**

A total of 102 patients without comorbidities and with systolic pressure (SP) < 120 mmHg and diastolic pressure (DP) < 80 mmHg were selected from the EVOPIU database (Pulse Wave Velocity of Elderly Individuals in an Urban area of Brazil). The carotid-femoral pulse wave velocity (c-fPWV) and the central and peripheral pressures were evaluated in all patients. The patients were divided into four groups: G1: (n = 19, with c-fPWV < 7.6 m/s, without medication), G2 (n = 26, c-fPWV ≥ 7.6 m/s; without medication), G3 (n = 25, c-fPWV < 7.6 m/s with antihypertensive medication), and G4 (n = 32, c-fPWV ≥ 7.6 m/s with antihypertensive medication).

**Results:**

In our sample, 56.7% of patients had c-fPWV ≥ 7.6 m/s. The central systolic pressure in G1 [99 (10) mmHg] was lower than that found in the other three groups [vs. 112 (14) mmHg, 111 (15), 112 (20) mmHg; P < 0.05)].

**Conclusion:**

Older people with optimal arterial blood pressure do not necessarily have HVA and could have c-fPWV values close to the limits established for CAS diagnosis.

## RESUMO

**Introdução:**

A velocidade da onda de pulso é usada para diagnosticar a rigidez arterial central (RAC) e quantificar o envelhecimento vascular saudável (EVS).

**Objetivo:**

Avaliar a RAC e o EVS em pacientes idosos com níveis pressóricos sistêmicos classificados como ideais/normais.

**Métodos:**

Um total de 102 pacientes sem comorbidades e com pressão sistólica (PS) < 120 mmHg e pressão diastólica (PD) < 80 mmHg foram selecionados do banco de dados EVOPIU (Estudo da Velocidade de Onda de Pulso em Idosos em área Urbana no Brasil). Foram avaliadas a velocidade da onda de pulso carotídeo-femoral (VOPcf) e as pressões central e periférica em todos os pacientes. Os pacientes foram divididos em quatro grupos: G1: (n = 19; com VOPcf < 7,6 m/s; sem medicação), G2 (n = 26; VOPcf ≥ 7,6 m/s; sem medicação), G3 (n = 25; VOPcf < 7,6 m/s com medicação anti-hipertensiva), e G4 (n = 32; VOPcf ≥ 7,6 m/s com medicação anti-hipertensiva).

**Resultados:**

Em nossa amostra, 56,7% dos pacientes apresentaram VOPcf ≥ 7,6 m/s. A pressão sistólica central no G1 [99 (10) mmHg] foi inferior à encontrada nos outros três grupos [vs. 112 (14) mmHg, 111 (15), 112 (20) mmHg; P < 0,05)].

**Conclusão:**

Pessoas idosas com pressão arterial ideal não necessariamente têm EVS e podem apresentar valores de VOPcf próximos aos limites estabelecidos para o diagnóstico de RAC.

## Introduction

Aging is one of the most important causes of central arterial stiffness (CAS) in elderly individuals. Central vessel stiffness is a risk factor for cardiovascular morbidity and mortality^1,2^. Systemic arterial hypertension (SAH) is the most prevalent disease among elderly individuals. The global prevalence of hypertension in the elderly is estimated to be approximately 1 billion individuals^3^.

The *Systolic Blood Pressure Intervention Trial* (SPRINT, 2015) showed that systolic pressures (SP) < 120 mmHg and diastolic pressures (DP) < 80 mmHg in elderly patients reduced cardiovascular risk by 25%, with lower rates of fatal and nonfatal events and death from any etiology^4^. The SPRINT results have changed the pressure targets in treating hypertension worldwide^5,6^. The *Brazilian Guidelines for Arterial Hypertension* began to classify patients as having optimal pressure with SP less than 120 mmHg and DP less than 80 mmHg^5^, while the American *Guideline for the Prevention, Detection, Evaluation, and Management of High Blood Pressure in Adults* classified the same systemic blood pressure (SBP) levels as normal^6^.

It has long been thought that hypertension leads to a thickening and stiffening of central arteries (i.e., stiffening is a consequence), whereas more recent evidence suggests that stiffening precedes hypertension (i.e., stiffening is a cause)^7^. Measurement of CAS by pulse wave velocity (PWV) has been suggested as an additional test to calculate cardiovascular risk in hypertensive patients^8^ and for adapting therapeutic strategies^9^. However, routine measurement of PWV is not practical and is not recommended for routine practice. On the other hand, PWV may be considered a physiological method for quantifying healthy arterial aging (HVA)^10^. The prevalence, correlates, and prognosis of HVA in elderly are incompletely understood. Our study aimed to verify HAV and CAS in elderly patients with systemic blood pressure (SBP) levels classified as optimal/normal.

## Method

The present study is a cross-sectional analysis for CAS evaluation in elderly patients classified as having optimal pressure from the database Study of Pulse Wave Speed in Elderly in Urban Area in Brazil (EVOPIU)^11^. The EVOPIU database consists of 1,204 patients over 60 years of age, with biannual clinical and laboratory examinations. The carotid-femoral wave velocity (c-fPWV) was measured each visit. The EVOPIU study lasted 48 months (from 2014 to 2018).

### Inclusion Criteria

The inclusion criteria were patients who presented at the initial EVOPIU visit with optimal systemic blood pressure levels according to the Brazilian Guidelines for Arterial Hypertension 2020^5^ and normal blood pressure according to the American Guidelines for Hypertension^6^. Both cutoffs are SP < 120 mmHg and DP < 80 mmHg.

### Exclusion Criteria

Patients with diabetes mellitus (DM), diagnosed by fasting glucose > 100 mg/dL or < 100 mg/dL while taking oral hypoglycaemic agents and/or insulin, and all patients with systemic blood pressure (SBP) above 120/80 mmHg were excluded from the study.

### Characterization of the Groups and Data Collection

According to inclusion and exclusion criteria, 102 patients were selected for the present study, representing 8.6% of the database. All patients underwent applanation tonometry to evaluate c-fPWV and were subsequently classified according to the values obtained for c-fPWV and whether or not they were using antihypertensive drugs.

We defined HVA as individuals having c-fPWV of < 7.6 m/s, optimal or normal blood pressures, and no additional cardiovascular risk factor^10^. For diagnosis of CAS, we used cut-off values of Mendonça et al., who calculated the values of c-fPWV for hypertensive and normotensive elderly in Brazil^11^.

**Figure 1.**
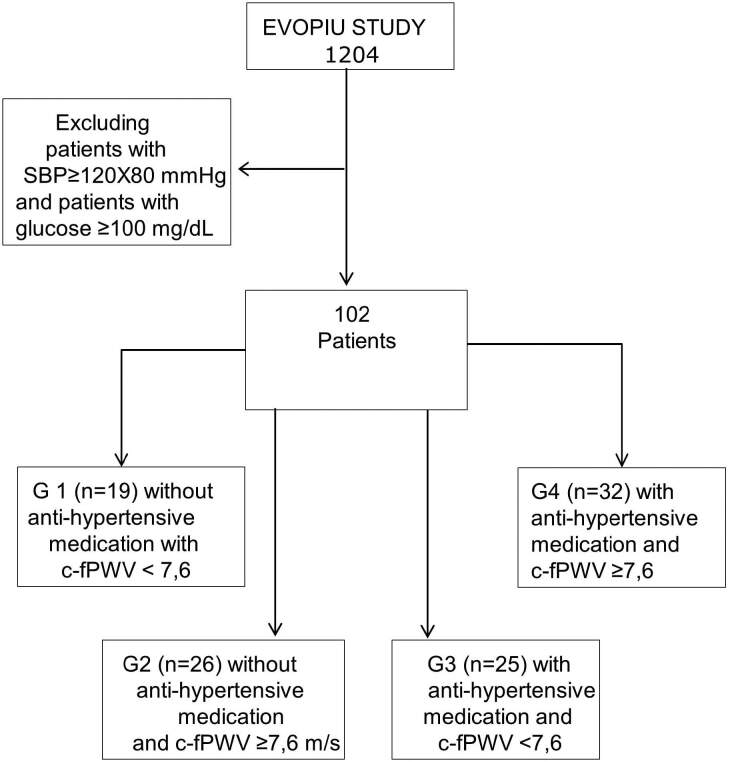
Study Design.

The patients were divided into four groups: G1 (n = 19), without antihypertensive drugs and c-fPWV < 7.6 m/s; G2 (n = 26), without antihypertensive drugs and c-fPWV ≥ 7.6 m/s; G3 (n = 25), using antihypertensive drugs and c-fPWV < 7.6 m/s; and G4 (n = 32), using antihypertensive drugs and c-fPWV ≥ 7.6 m/s (Figure 1). Brachial systemic blood pressure (bSBP) was measured after 10 minutes of rest, in triplicate, in the sitting position, with 3-min intervals between measurement with an automatic digital blood pressure oscillometric device (HE 7200 *Intelli Sense Omron Hem* ^®^, Brazil). The values used are the arithmetic mean of the three measurements in mmHg. The cuffs of the devices were calibrated and adapted to the circumference of the arms of the participants. Serum levels of uric acid, urea, and creatinine, blood glucose, and lipid profile were assessed using colorimetric methods (Cobas^®^ 6000; Roche Hitachi, Brazil), whereas hematological examination was performed with a Sysmex^®^ XED-2100 (São Paulo, Brazil). Glomerular filtration rate (eGFR) was estimate by the CKD-EPI equation^12^.

### Aplanation Tonometry – Central Blood Pressure, c-fPWV and AIx

Applanation tonometry (AT) was performed with the SphygmoCor^®^ XCEL device, model EM4C (AtCor Medical, Sydney, AU), which measured: the central and peripheral systolic (cSP, bSP) and diastolic pressure (cDP, bDP), pulse pressure (cPP, bPP), mean arterial pressure (cMAP, bMAP), amplification of arterial pulse pressure (AP), augmentation index (Aix %), heart rate (HR) and c-fPWV (m/s).

### Statistical Analysis

Simple frequency descriptive analyses were performed for the variables, with measures of central tendency (mean and median) and variability (standard deviation and interquartile range). Data were collected in electronic spreadsheets, and statistical tests were performed using Stata software version 17.

After analyzing the assumptions of normality by the Shapiro-Wilk test, ANOVA tests were applied for the parametric variables and Kruskal-Wallis tests were applied for the nonparametric variables. The c-fPWV values were adjusted for sex, age, and bMAP (c-fPWV adj.) (m/s). A value of p < 0.05 was considered an indication of statistical significance.

## Results

The clinical and laboratory characteristics of the evaluated patients are shown in Table 1. The data for brachial and central SPB and the data obtained by the application of tonometry are shown in Table 2. The medications used by each group are listed in Table 3.

**Table 1.** Distribution of anthropometric and laboratory data by groups

	Groups n (102) (%)			
Variables	1	2	3	4
	n: 19 (18.6)	n: 26 (25.4)	n: 25 (24.5)	n: 32 (31.3)
Age (years)	65 (6)	66 (11)	64 (4)^d^	71 (10)^cf^
Height (m)	1.54 (0.12)	1.59 (0.14)	1.54 (0.07)^d^	1.60 (0.14)^f^
Sex (%) Female	16 (84)	16 (62)	24 (84)	16 (50)
Weight (kg)	60.0 (15.4)	62.95 (16.0)	60.0 (13.5)	70.5 (16.3)^cef^
AC (cm)	89.4 ± 13.8	88.2 ± 13.4	94.8 ± 7.5^b^	96.3 ± 12.6^ce^
HR (bpm)	71.6 ± 4.8	70.1 ±12.0	76.4 ± 10.9	74.8 ± 14.6
BMI (kg/m^2^)	24 (6.4)	26 (8.0)	25 (3.4)	28 (5.6)
**Laboratory mg/dL**				
TC	203 (39.6)	203 (58.0)	177.5 (32.2)	183.5 (65.0)
HDL	52 (11)	53 (17)	45 (27)	45 (16)
LDL	125.4 (54.4)	121.0 (44.9)	103.5 (27.7)^bd^	110.2 (49.0)
Tg	108 (59)	100 (37)	123 (79)	121 (99)
Glu	87 (12.9)	84 (13)	88 (10.5)	91 (7.1)
UrA	4.1 (1.9)	5.1(1.4)^a^	5.1 (2.7)^b^	6.0 (2.1)^ce^
Cr	0.6 (0.1)	0.7 (0.2)^a^	0.8 (0.2)^b^	0.9 (0.4)^cef^
eGFR	94 ± 11.1	86 ± 12.8	89 ± 23.1	70 ± 18.8^cef^
Hb (%)	41 (3.6)	41 (3.7)	41 (4.5)	40 (4.4)
Hct (g/%)	13.6 (1.2)	13.6 (1.2)	13.7 (1.4)	13.4 (1.5)

*P*< 0.05; a: 1 vs 2, b: 1 vs 3, c: 1 vs 4, d: 2 vs 3, e: 2 vs 4, f: 3 vs 4; Abdominal Circumference (AC); Heart Rate (HR); Body Mass Index (BMI); Total cholesterol (TC); High Density Lipoproteins (HDL); Low Density of Lipoproteins (LDL); Triglycerides (Tg); Blood Glucose (Glu); Hemoglobin (Hb); Hematocrit (Hct.); Uric Acid (Ur.A); Creatinine (Cr.), GFR: glomerular filtration rate (ml/min/m^2^) calculate by CKD-EPI.

**Table 2.** Blood pressure and applanation tonometry data by groups

	Groups n (102)			
Variables	1	2	3	4
	n: 19	n: 26	n: 25	n: 32
**Systemic Blood Pressure mmHg**				
bSP	109 (13.0)	116 (7.0)^a^	112 (8.0)	115 (6.5)^c^
bDP	68 (9)	70 (7)	69 (10)	71 (8)
bPP	41.7 ± 5.1	45.4 ± 5.5	43.5 ± 6.9	44.1 ± 5.7
bMAP	81.6 (9.4)	84.3 (6.3)	83.0 (7.1)	84.8 (6.1)
cPS	99 (10)	112 (14)^a^	111 (15)^b^	112 (20)^c^
cPD	69 (10)	72 (10)	72 (9)	75 (14)
cPP	35 (10)	35 (9)	39 (10)	38 (14)
cMAP	82 (11)	89 (11)	87 (10)	93 (17)^c^
**Applanation Tonometry**				
PA (mmHg)	14.0 ± 6.8	12.7 ± 7.6	14.8 ± 7.3	15.4 ± 8
AIX (%)	38.0 (15.4)	30.8 (18.5)	38.5 (13.5)	37.4 (12.4)
c-fPWV m/s	6.5 (1.4)	8.7 (1.9)^a^	6.5 (1.1)	8.9 (2.1)^c^
c-fPWV Adj. (m/s)	6.7± 0.31	9.1 ± 0.26^a^	6.7 ± 0.27^d^	9.1 ± 0.25^cf^

*P*< 0.05; a: 1 vs 2, b: 1 vs 3, c: 1 vs 4, d: 2 vs 3, e: 2 vs 4, f: 3 vs 4; Brachial systolic pressure (bSP); Brachial Diastolic Pressure (bDP); Brachial Pulse Pressure (bPP); brachial Mean Arterial Pressure (bMAP); Central Systolic Pressure (cSP); Central Diastolic Pressure (cDP); Central Pulse Pressure (cPP); central Mean Arterial Pressure (cMAP); Incremental Pressure (AP); Augmentation Index (AIx); Carotid-femoral Pulse wave velocity (c-fPWV); Adjusted pulse wave velocity (c-fPWV adj).

**Table 3.** Oral drugs by groups

	Groups n (102)			
Variables	1	2	3	4
	n: 19	n: 26	n: 25	n: 32
**Medication n (%)**				
Diuretics	0	0	21 (84)^bd^	24 (75)^ce^
Beta blockers	0	0	9 (36)^bd^	11 (34)^ce^
BCC	0	0	4 (16)^bd^	5 (15)^ce^
Vasodilators	0	0	1 (4)^bd^	1 (3)^ce^
ACEI	0	0	12 (48)^bd^	15 (46)^ce^
ARB	0	0	9 (36)^bd^	12 (37)^ce^
Statin	4 (21)	2 (7)	9 (36)	8 (25)
NSAIDs	1 (5)	0	8 (32)	10 (31)
Antiulcer	1 (5)	3 (11)	5 (20)	0
Insulin	0	0	0	0
HO	0	0	0	0

P < 0.005 = a = 1 vs 2, b = 1 vs 3, c = 1 vs 4, d = 2 vs 3, e = 2 vs 4 f = 3 vs 4; Beta-blockers (BB); Calcium Channel Blockers (BCC); Angiotensin-converting enzyme inhibitors (ACEI); Angiotensin receptor blockers (ARB); Nonsteroidal anti-inflammatory (NSAIDs) Oral Hypoglycaemic (HO).

The mean age of the patients was 67.8 ± 6.5 years. The median age was similar among the groups. Regarding sex, 70.6% of the patients were women, a similar percentage in each of the four groups. There was a statistically significant difference in the body weight among groups, being higher in G4 (*P*< 0.05), and the body mass index (BMI) did not differ among groups. There were no differences among groups for blood glucose, hemoglobin, hematocrit, uric acid, triglycerides, cholesterol, and HDL values. The serum creatinine values differed in all groups and were higher in G4.

The brachial blood pressures were similar in all groups, except in G1, and bSP was different in G2 and G4. The cSP values of G1 were the lowest of the four groups.

**Figure 2.**
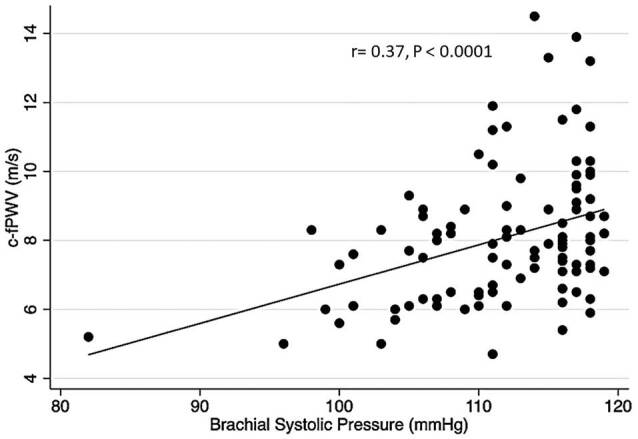
Correlation between c-fPWV and bSP in elderly individuals with optimal blood pressure.

The adjusted c-fPWV in G1 was 6.7 ± 0.31 m/s and was lower than groups G2 and G4 (*P*< 0.005) and similar with G3. The adjusted c-fPWV in G2 and G4 were similar. G1 and G2 did not use antihypertensive while G3 and G4 used them regularly. The correlations between c-fPWV, bSP, cSP, bPP, and cPP were calculated, and the following results were obtained: c-fPWV and bSP (r = 0.37, *P*< 0.0001), c-fPWV and cSP (r = 0.29, P < 0.0029), c-fPWV and bPP (r = 0.19*, P*= 0.052, NS), c-fPWV and cPP (r = 0.11, *P*= 0.261, NS). The correlation between c-fPWV and bPS is shown in Figure 2.

## Discussion

This study showed that 18.6% of the total sample of elderly had optimal pressure (OP), no comorbidities, and with c-fPWV within the parameters considered good vascular health (G1). The elderly individuals in this group did not have DM or other comorbidities and did not use antihypertensive medications. Group G2 (optimal pressure without reported comorbidities) showed an increase in c-fPWV at levels compatible with the cut-off of CAS diagnosis for hypertensive patients^11^. Although CAS has been considered a complication of hypertension, there is increasing evidence that arterial stiffness may precede the increase in SBP, and an increase in bSP further increases arterial stiffness^13, 14, 15, 16^.

The antihypertensive drugs used by G3 and G4 and c-fPWV are shown in Table 3. Despite using antihypertensives of similar classes, the c-fPWV of group G3 was lower than that observed in G4, 6.5 (1.1) m/s vs. 8.9 (2.1) m/s, *P*< 0.05. The treatment did not seem to normalize c-fPWV in all hypertensive patients or the efficacy of hypotensive drugs on the stiffness of the arterial wall was not yet evidenced in G4.

Qu, Zhang, and Zhu^17^ studied the arterial stiffness of hypertensive patients with and without DM in patients aged 45 to 97 years and found a positive correlation between SAH and the severity of arterial vessel thickening. The authors found that patients with uncontrolled SAH had higher arterial stiffness than those with controlled SAH. However, our data showed that elderly patients, even those with OP, had c-fPWV at the limits of CAS (G2, G4). Figure 1 shows that even for elderly patients with OP, there is a weak and significant positive correlation between c-fPWV and bSP; that is, as systolic pressure increases, c-fPWV also increases in those with optimal pressure. However, the studies have some methodological differences, including nonelderly individuals, the presence of DM, the method of measuring c-fPWV, and higher SBP levels.

Our data indicate that OP in elderly individuals does not necessarily mean compliant central arterial vessels or HVA. However, the Consensus of the European Society of Hypertension/European Society of Cardiology^8^ recommends assessing subclinical damage in target organs only in hypertensive patients. The present study demonstrates that, despite OP, the c-fPWV values may be high and exceed the limits defined for HVA (G2,G4). Some antihypertensives, such as spironolactone^18^, calcium channel blockers^19^, and inhibitors of the renin-angiotensin system, may reduce CAS regardless of SBP decrease^20, 21, 22, 23^ or its hypotensive associations^24^ related to the reduction of CAS regardless of SBP levels.

In a study by Freitas *et al*.^25^, the authors concluded that patients with good vascular health are more protected against occasional SBP elevations than other groups without these conditions. In our study, it is important to note that c-fPWV was correlated with brachial and central systolic pressures, while pulse pressures were not correlated. In elderly individuals, bSP and bPP are related to CAS^26, 27, 28^; however, bPP was not correlated with c-fPWV in elderly individuals with OP. There is a possibility that this relationship becomes evident with higher SBP levels. Vatner *et al*.^28^ demonstrated that arterial stiffness is linearly related to age, both in normotensive and severely hypertensive individuals. Safar *et al*.^29^ showed that the slopes of these linear relationships are not different; in other words, arterial stiffness increase in normotensive individuals in the same way as in hypertensive individuals. Figure 1 shows that this also occurs in elderly individuals with OP.

CAS plays an important role in increasing microvascular pulsatility with consequent glomerular injury^30^. The serum creatinine levels of G4 differed from those of the other groups (Table 1). G4 had increased creatinine and reduced glomerular filtration rate, and they coexisted with an increase in c-fPWV compared to G3.

### Study Limitations

The present was cross-sectional study, with the limitations inherent to this type of design. The small number of patients in certain groups may not be allow data extrapolation to larger populations. The study did not analyze the duration of arterial hypertension and the consequent increase in arterial stiffness and did not evaluate the effect of hypotensive drugs on c-fPWV. Under these conditions, some patients with c-fPWV within normal parameters could present only one evolutionary phase of the disease.

## Conclusions

Older people with optimal pressure do not necessarily have HVA and may have c-fPWV values close to the limits established for CAS diagnosis.
